# An electrographic AV optimization for the maximum integrative atrioventricular and ventricular resynchronization in CRT

**DOI:** 10.1186/s12872-021-02096-1

**Published:** 2021-06-10

**Authors:** Jie Li, Yuegang Wang, Jingting Mai, Shilan Chen, Menghui Liu, Chen Su, Xumiao Chen, Huiling Huang, Yuedong Ma, Chong Feng, Jingzhou Jiang, Jun Liu, Jiangui He, Anli Tang, Yugang Dong, Xiaobo Huang, Yangxin Chen, Lichun Wang

**Affiliations:** 1 Department of Cardiology, The First Affiliated Hospital of Sun Yat-Sen University, No. 58, Zhongshan 2nd Rd, Guangzhou, 510080 Guangdong People’s Republic of China; 2 Key Laboratory On Assisted Circulation, Ministry of Health, Guangzhou, People’s Republic of China; 3 Department of Cardiology, Sun Yat-Sen Memorial Hospital of Sun Yat-Sen University, No.107, Yanjianxi Rd, Guangzhou, 510080 Guangdong People’s Republic of China; 4 Department of Cardiology, Nanfang Hospital of Southern Medical University, Guangzhou, People’s Republic of China

**Keywords:** AV delay, Atrioventricular synchrony, Ventricular synchrony, Cardiac resynchronization therapy

## Abstract

**Background:**

Atrioventricular (AV) delay could affect AV and ventricular synchrony in cardiac resynchronization therapy (CRT). Strategies to optimize AV delay according to optimal AV synchrony (AV_opt-AV_) or ventricular synchrony (AV_opt-V_) would potentially be discordant. This study aimed to explore a new AV delay optimization algorithm guided by electrograms to obtain the maximum integrative effects of AV and ventricular resynchronization (opt-AV).

**Methods:**

Forty-nine patients with CRT were enrolled. AV_opt-AV_ was measured through the Ritter method. AV_opt-V_ was obtained by yielding the narrowest QRS. The opt-AV was considered to be AV_opt-AV_ or AV_opt-V_ when their difference was < 20 ms, and to be the AV delay with the maximal aortic velocity–time integral between AV_opt-AV_ and AV_opt-V_ when their difference was > 20 ms.

**Results:**

The results showed that sensing/pacing AV_opt-AV_ (SAV_opt-AV_/PAV_opt-AV_) were correlated with atrial activation time (P_end-As_/P_end-Ap_) (*P* < 0.05). Sensing/pacing AV_opt-V_ (SAV_opt-V_/PAV_opt-V_) was correlated with the intrinsic AV conduction time (As-Vs/Ap-Vs) (*P* < 0.01). The percentages of patients with more than 20 ms differences between SAV_opt-AV_/PAV_opt-AV_ and SAV_opt-V_/PAV_opt-V_ were 62.9% and 57.1%, respectively. Among them, opt-AV was linearly correlated with SAV_opt-AV_/PAV_opt-AV_ and SAV_opt-V_/PAV_opt-V._ The sensing opt-AV (opt-SAV) = 0.1 × SAV_opt-AV_ + 0.4 × SAV_opt-V_ + 70 ms (R^2^ = 0.665, *P* < 0.01) and the pacing opt-AV (opt-PAV) = 0.25 × PAV_opt-AV_ + 0.5 × PAV_opt-V_ + 30 ms (R^2^ = 0.560, *P* < 0.01).

**Conclusion:**

The SAV_opt-AV_/PAV_opt-AV_ and SAV_opt-V_/PAV_opt-V_ were correlated with the atrial activation time and the intrinsic AV conduction interval respectively. Almost half of the patients had a > 20 ms difference between SAV_opt-AV_/PAV_opt-AV_ and SAV_opt-V_/PAV_opt-V_. The opt-AV could be estimated based on electrogram parameters.

## Background

Cardiac resynchronization therapy (CRT) is a milestone therapy in advanced congestive heart failure for its ability in decreasing symptoms, improving quality of life and exercise capacity, and reducing hospitalization and mortality in selected patients with heart failure [1, 2]. However, up to 30–45% of patients do not respond to CRT therapy [1, 3]. Among them, almost 50% cases have suboptimal atrioventricular (AV) timing [4].

The AV interval can affect AV and ventricular synchrony simultaneously. However, improving AV and ventricular dyssynchrony is the underlying therapeutic mechanism of CRT. To obtain optimal AV synchrony, an AV interval is required to ensure that the left ventricle (LV) only contracts after completion of left atrial (LA) contraction [5]. At this time, the optimal AV interval is subject to the atrial activation time. If there is an inter/intra-atrial conduction delay, a relatively long AV interval is required for delayed LA contraction. Furthermore, ventricular resynchronization is maximally achieved through the narrowest QRS, which is obtained by optimal fusion between intrinsic atrioventricular activation and paced activation [6, 7]. Therefore, the optimal AV interval should coincide with the intrinsic AV conduction interval for obtaining optimal ventricular synchrony.

As a result, in some cases, the optimal AV delay for maintaining AV synchrony may be quite different from that for maintaining ventricular synchrony. For example, in patients with a long PR interval but normal atrial conduction, optimal ventricular resynchronization (the narrowest QRS) would require a longer AV delay, while this might lead to suboptimal AV resynchronization since a normal atrial conduction requires a relatively short AV delay.

In this study, the AV intervals were optimized according to optimal AV synchrony (AV_opt-AV_) and optimal ventricular synchrony (AV_opt-V_). The relationships between AV_opt-AV_ and the atrial activation time, AV_opt__-V_ and the intrinsic AV interval, AV_opt-AV_ and AV_opt-V_ were further investigated. The aim was to study the difference between AV_opt-AV_ and AV_opt-V_, and to propose a novel AV optimized algorithm guided only by the intrinsic AV interval and the atrial activation time to obtain the maximal integrative effects of AV and ventricular resynchronization.

## Methods

### Study population

This was a multicenter, nonrandomized study, that enrolled patients aged 18 or older, who had been implanted with CRT defibrillators with standard criteria (NYHA classes II-IV; ejection fraction ≤ 35%; sinus rhythm; left bundle branch block with QRS ≥ 130 ms). Patients were excluded from the study if they had congenital heart diseases, valve repair or replacement surgeries, atrial tachyarrhythmias or frequent atrial or ventricular ectopy, and second/third degree of AV block. The measurements were performed at least 1 month after CRT implantation to reduce the effect of the operation. The protocol of this study was approved by the institutional review boards of the participating hospitals, and all patients gave written informed consent.

### Study procedures

#### General data

Clinical data, such as demographics (age, sex, etc.), etiology, heart failure status before CRT device implantation (NYHA classes, left ventricular end-diastolic diameter, left ventricular end-systolic diameter, ejection fraction), medications, CRT device data (model number and date of implant), and the location of the right atrium and LV electrodes were collected before the study procedure.

#### Electrocardiogram and device electrograms

**Fig. 1:**
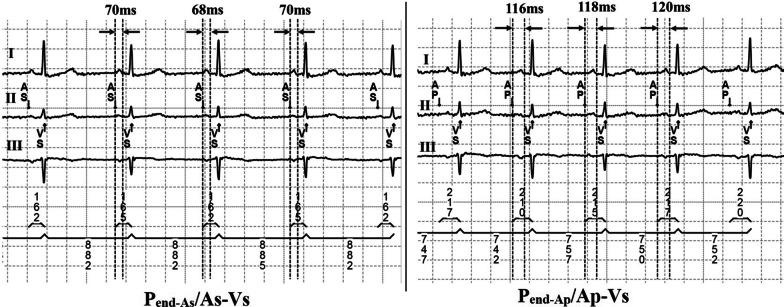
Measurement of atrial activation time and intrinsic atrioventricular conduction time. Left panel: atrial activation time (P_end-As_) and intrinsic atrioventricular conduction time (As-Vs) at the time of atrial sensing. Right panel: atrial activation time (P_end-Ap_) and intrinsic atrioventricular conduction time (Ap-Vs) at the time of atrial pacing. A_S_: atrial sensing; A_P_: atrial pacing; V_S_: ventricular sensing

The patient’s intrinsic electrocardiogram (ECG) was recorded after the device marker recording showed atrial sensing (As) and ventricular sensing (Vs) by programming the lower rate to 50/40 bpm and the sensed AV delay to 300/350 ms. Heart rate, QRS duration, PR interval, device-recorded intrinsic AV conduction interval (As-Vs), and atrial activation time (duration from As to the end of the P wave [P_end-As_]) were sequentially measured. Then the lower rate was programmed to 10 bpm higher than the intrinsic heart rate. The interval from atrial pacing (Ap) to Vs (Ap-Vs), and the duration from Ap to the end of the P wave (P_end-Ap_) were also measured sequentially at least 10 times (Fig. 1).

#### Measurement of AV delay according to the optimal atrioventricular synchrony

The Ritter method was originally developed for AV delay setting to achieve optimal AV synchrony in patients with a complete AV block and preserved LV function [8]. Its aim was to maximize LV filling (including maximizing the role of LA contraction) and to minimize pre-systolic mitral regurgitation by ensuring that the left ventricular contraction starts soon after the completion of the left atrial contraction. Then, it and its analogue became the gold standard for AV delay optimization in CRT [9]. In this study, we used the Ritter method to optimize AV_opt-AV_. In brief, mitral flow velocity and surface ECG were simultaneously recorded. A short and a long AV delay (AV_short_/AV_long_) were programmed, and the relevant intervals from the pacing spike (Q) to the end of the Doppler mitral inflow A wave (QA_short_/QA_long_) were measured. AV_opt-AV_ was calculated as follows: AV_opt-AV_ = AV_long_ + QA_long_ − QA_short_ [8, 10]. In this study, the VV intervals were always kept at 0 ms.

#### Measurement of the AV delay according to the optimal ventricular synchrony

**Fig. 2:**
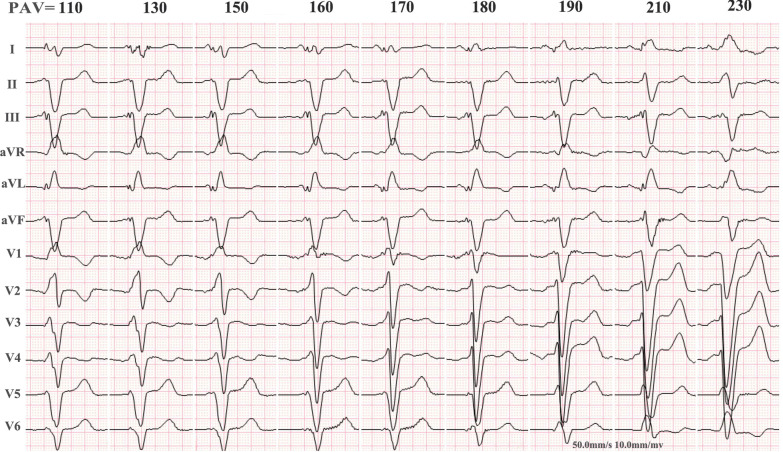
Measurement of atrioventricular delay according to the optimal ventricular resynchronization. A representative electrocardiographic series shows the measurement of pacing atrioventricular delay for optimal ventricular resynchronization. Ap-Vs = 260 ms, pacing rate = 90 bpm. The numbers shown on the top of the electrocardiograms are the values (ms) of PAV. The narrowest QRS (128 ms) occurred at a PAV of 170 ms. Ap-Vs: the intrinsic atrioventricular conduction time at the time of atrial pacing. PAV: pacing AV delay

The narrower the QRS duration was, the more synchronous the ventricle was. Simultaneous 12-lead ECGs were recorded when the sensing/pacing AV delays (SAV/PAV) were programmed to values from 70 ms to an AV delay of 40 ms less than the intrinsic As-Vs/Ap-Vs by 10-ms steps in a random order. The durations of the QRS were automatically calculated by the built-in software in the ECG machine. The SAV/PAV delays with the narrowest QRS duration were considered sensing/pacing AV_opt-V_ (SAV_opt-V_/PAV_opt-V_) (Fig. 2).

#### Determination of the optimal AV delay

When sensing/pacing AV_opt-AV_ (SAV_opt-AV_/PAV_opt-AV_) with SAV_opt-V_/PAV_opt-V_ was compared, if the difference in values (D-values) was < 20 ms, SAV_opt-AV_/PAV_opt-AV_ and SAV_opt-V_/PAV_opt-V_ were regarded as not different [11, 12], and were considered as the optimal sensing/pacing AV delay (opt-SAV/opt-PAV). If the D-values were ≥ 20 ms, the aortic velocity–time integral (AoVTI) was measured by continuous wave Doppler recordings from SAV_opt-AV_/PAV_opt-AV_ to SAV_opt-V_/PAV_opt-V_ by 10-ms steps. SAV/PAV with the maximum AoVTI was considered as the opt-SAV/opt-PAV. All measurements of echocardiographic data were averaged from 9 to 12 consecutive cardiac beats.

#### Statistical analysis

Continuous variables that were normally distributed are shown as the mean ± SD. Non-normally distributed variables are shown as medians and interquartile ranges. The data were analyzed with IBM SPSS software version 20.0 for Windows (IBM Inc., Armonk, NY, USA). The paired-samples t-test or two related-samples Wilcoxon rank sum tests were used for between-group statistical analysis according to the evaluation of a normal distribution. Regression analysis and Pearson’s correlation coefficient were performed to evaluate the correlations. *P* < 0.05 was defined as statistically significant.

## Results

### Patient population

A total of 49 patients were enrolled in this study from three hospitals (the First Affiliated Hospital of Sun Yat-Sen University, Sun Yat-Sen Memorial Hospital of Sun Yat-Sen University, and Nanfang Hospital of Nanfang Medical University) from July 2017 to May 2020. The clinical characteristics of the patients are shown in Table 1. The majority of the patients (35/49) were men. The mean intrinsic PR interval and QRS duration were 185.88 ± 38.20 (115–275) ms and 166.29 ± 21.34 (146–237) ms, respectively. The atrial electrodes were placed in the right atrial appendage in all patients.

**Table 1:** Clinical characteristics of the patients involved in the study

Age, years	67.25 ± 9.37
**Male/female**	35/14
**Etiology, n (%)**	
Ischemic heart disease	20 (40.8)
Dilated myocardiopathy	24 (49.0)
Noncompaction of ventricular myocardium	4 (8.2)
Other	1 (2.0)
**NYHA class, n (%)**	
II	13 (28.9)
III	29 (64.4)
IV	4 (8.9)
**Medication, n (%)**	
Beta-blocker	42 (93.3)
ACEI/ARB	27 (60.0)
MRA	44 (97.8)
ARNI	18 (40.0)
I_f_-channel inhibitor	3 (6.7)
Diuretics	4 (8.9)
Digitalis	3 (6.7)
**UCG**	
LV EF (%)	28.66 ± 4.94
LVEDD (mm)	70.42 ± 10.29
LVESD (mm)	59.13 ± 10.94
PA pressure (mmHg)	38.45 ± 6.98
Diastolic mitral regurgitation, n (%)	31 (68.9)
**ECG characteristics**	
Intrinsic heart rate (bpm)	68.21 ± 11.76
PR interval (ms)	185.88 ± 38.20
QRS duration (ms)	166.29 ± 21.34
**Location of LV lead, n (%)**	
Short axis	
Lateral/posterolateral	32 (65.3)
Anterolateral	13 (25.5)
Posterior	4 (8.2)
Anterior	0
Long axis	
Basic	16 (32.7)
Middle	33 (67.3)
Apical	0
**Device electrogram characteristics**	
As-Vs (ms)	200.02 ± 34.26
Ap-Vs (ms)	258.33 ± 45.46
P_end-As_ (ms)	86.08 ± 22.68
P_end-Ap_ (ms)	136.84 ± 23.92

NYHA: New York heart association; ACEI: Angiotensin-converting-enzyme inhibitor; ARB: Angiotensin II receptor antagonist; MRA: Aldosterone receptor antagonist; ARNI: Angiotensin receptor-neprilysin inhibitor; I_f_: Funny current; UCG: Ultrasonic cardiogram; LVEF: left ventricular ejection fraction; LVEDD: left ventricular end diastolic diameter; LVESD: left ventricular end systolic diameter; PA: Pulmonary artery; ECG: Electrocardiogram; As-Vs: Intrinsic atrioventricular conduction time at the time of atrial sensing; Ap-Vs: Intrinsic atrioventricular conduction time at the time of atrial pacing; P_end-As_: Atrial activation time at the time of atrial sensing; P_end-Ap_: Atrial activation time at the time of atrial pacing

### Relationship between AV_opt-AV_ and atrial activation time

AV_opt-AV_ was successfully determined in 35 patients by the Ritter method. Regression analysis showed that SAV_opt-AV_ was significantly correlated with P_end-As_ (SAV_opt-AV_ = 0.80 × P_end-As_ + 50 ms, R^2^ = 0.467, *P* < 0.01). A similar result was also found between PAV_opt-AV_ and P_end-Ap_ (PAV_opt-AV_ = 0.70 × P_end-Ap_ + 70 ms, R^2^ = 0.221, *P* < 0.05).

### Relationship between AV_opt-V_ and intrinsic AV conduction and QRS duration

SAV_opt-V_/PAV_opt-V_ was achieved through the narrowest QRS duration in all 49 patients. SAV_opt-V_/PAV_opt-V_ was significantly correlated with As-Vs/Ap-Vs (SAV_opt-V_ = 0.60 × As-Vs + 15 ms, R^2^ = 0.456, *P* < 0.01; PAV_opt-V_ = 0.60 × Ap-Vs + 40 ms, R^2^ = 0.417, *P* < 0.01), but there was no significant correlation with the intrinsic QRS duration (both *P* > 0.5).

### Difference between AV_opt-AV_ and AV_opt-V_

In the 35 patients, in which SAV_opt-AV_/PAV_opt-AV_ and SAV_opt-V_/PAV_opt-V_ were successfully directly measured simultaneously, no significant correlations were found between SAV_opt-AV_ and SAV_opt-V_, or between PAV_opt-AV_ and PAV_opt-V_ (both *P* > 0.05). If > 20-ms D-values between SAV_opt-AV_/PAV_opt-AV_ and SAV_opt-V_/PAV_opt-V_ were considered as meaningful differences, 22/35 (62.9%) patients had a > 20-ms difference in SAV and 20/35 (57.1%) had a > 20-ms difference in PAV. Among them, 17/35 (48.6%) patients had a > 20-ms difference in both SAV and PAV. Moreover, if SAV_opt-AV_/PAV_opt-AV_ and SAV_opt-V_/PAV_opt-V_ were obtained by calculation according to their relationship with P_end-As_/P_end-Ap_ and As-Vs/Ap-Vs, respectively, > 20-ms D-values were found in 22/49 (44.9%) patients in SAV, in 29/49 (59.2%) patients in PAV, and in 17/49 (34.7%) patients in both SAV and PAV (Table 2).

**Table 2:** The percentage of patients with more than 20 ms differences between AV_opt-AV_ and AV_opt-V_

	Patients with directly measured AV (n = 35)	Patients with calculated AV* (n = 49)
SAV	22 (62.9%)	22 (44.9%)
PAV	20 (57.1%)	29 (59.2%)
SAV and PAV	17 (48.6%)	17 (34.7%)

^*^The Sensing/Pacing AV_opt-AV_ and AV_opt-V_ were calculated according to the following formulae:

SAV_opt-AV_ = 0.80 × P_end-As_ + 50 ms

SAV_opt-V_ = 0.60 × As-Vs + 15 ms

PAV_opt-AV_ = 0.70 × P_end-Ap_ + 70 ms

PAV_opt-V_ = 0.60 × Ap-Vs + 40 ms

### Relationships of opt-SAV/opt-PAV with SAV_opt-AV_/PAV_opt-AV_ and SAV_opt-V_/PAV_opt-V_

**Fig. 3:**
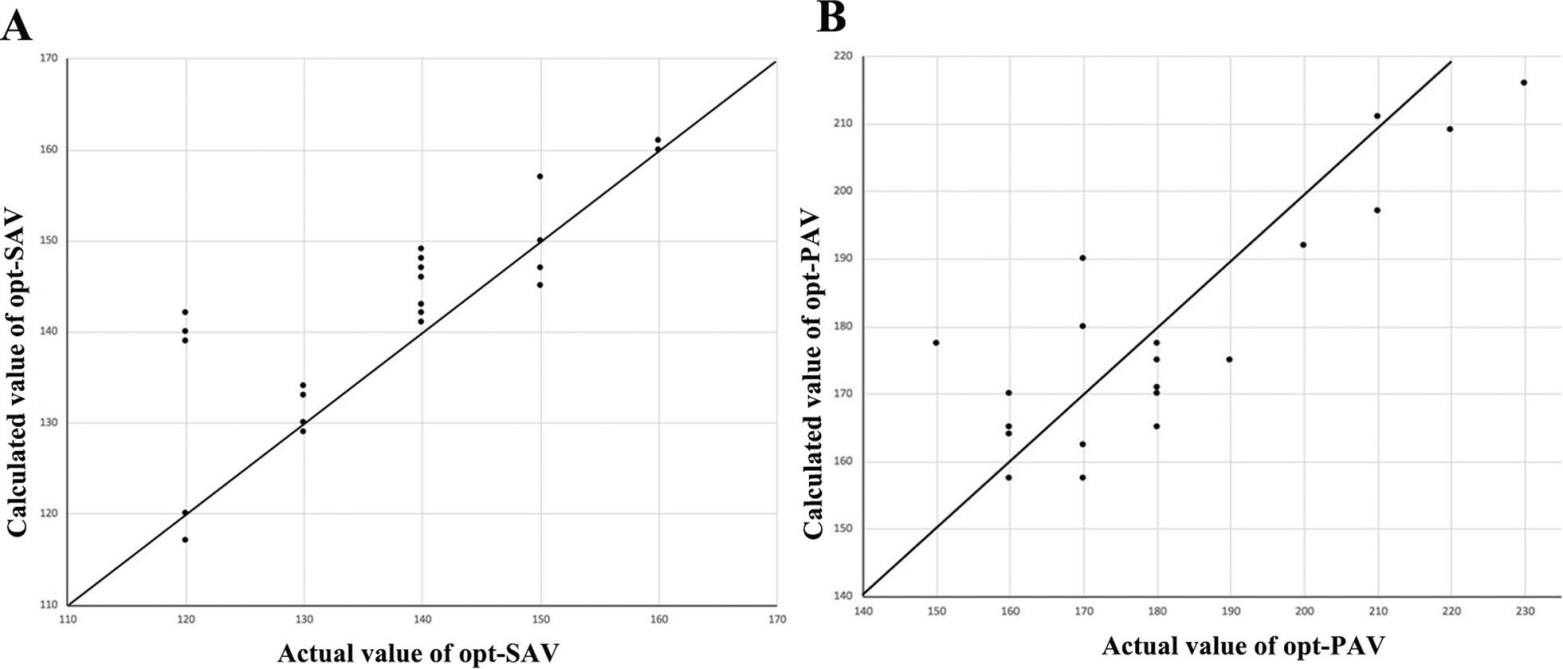
The relationship between actual opt-SAV/opt-PAV and the calculated opt-SAV/opt-PAV. The scatter plots between actual opt-SAV and the calculated opt-SAV (**a**) and between actual opt-PAV and the calculated opt-PAV (**b**). Actual opt-SAV/opt-PAV was the SAV/PAV with the maximum aortic velocity–time integral. Calculated opt-SAV = 0.1 × SAV_opt-AV_ + 0.4 × SAV_opt-V_ + 70 ms (n = 22, R^2^ = 0.665, *P* < 0.01); calculated opt-PAV = 0.25 × PAV_opt-AV_ + 0.5 × PAV_opt-V_ + 30 ms (n = 20, R^2^ = 0.560, *P* < 0.01)

Opt-SAV/opt-PAV was considered according to the maximum AoVTI when the D-values between SAV_opt-AV_/PAV_opt-AV_ and SAV_opt-V_/PAV_opt-V_ were > 20 ms. Regression analysis showed that opt-SAV = 0.1 × SAV_opt-AV_ + 0.4 × SAV_opt-V_ + 70 ms (R^2^ = 0.665, *P* < 0.01) and that opt-PAV = 0.25 × PAV_opt-AV_ + 0.5 × PAV_opt-V_ + 30 ms (R^2^ = 0.560, *P* < 0.01). The relative higher coefficient of determination (R^2^) in these equations indicated that the actual values and the calculated values of the opt-SAV/opt-PAV were highly related (Fig. 3).

### Echocardiographic evaluation of opt-SAV/opt-PAV

The difference in haemodynamics was evaluated by echocardiographic measurement of AoVTI. In the cases with > 20 ms D-values between SAV_opt-AV_ and SAV_opt-V_, the AoVTI on opt-SAV was significantly greater than the AoVTI on SAV_opt-v_ (opt-SAV − SAV_opt-V_ = 1.52 ± 0.22 cm, *P* < 0.001), and was not less than that on SAVopt-AV (opt-SAV − SAV_opt-AV_ = 0.89 ± 0.82 cm, *P* = 0.290). Furthermore, the AoVTI on opt-PAV was also significantly greater than that on PAV_opt-AV_ (opt-PAV − PAV_opt-AV_ = 2.47 ± 0.80 cm, *P* = 0.006) and PAV_opt-V_ (opt-PAV − PAV_opt-V_ = 0.76 ± 0.30 cm, *P* = 0.021).

## Discussion

**Fig. 4:**
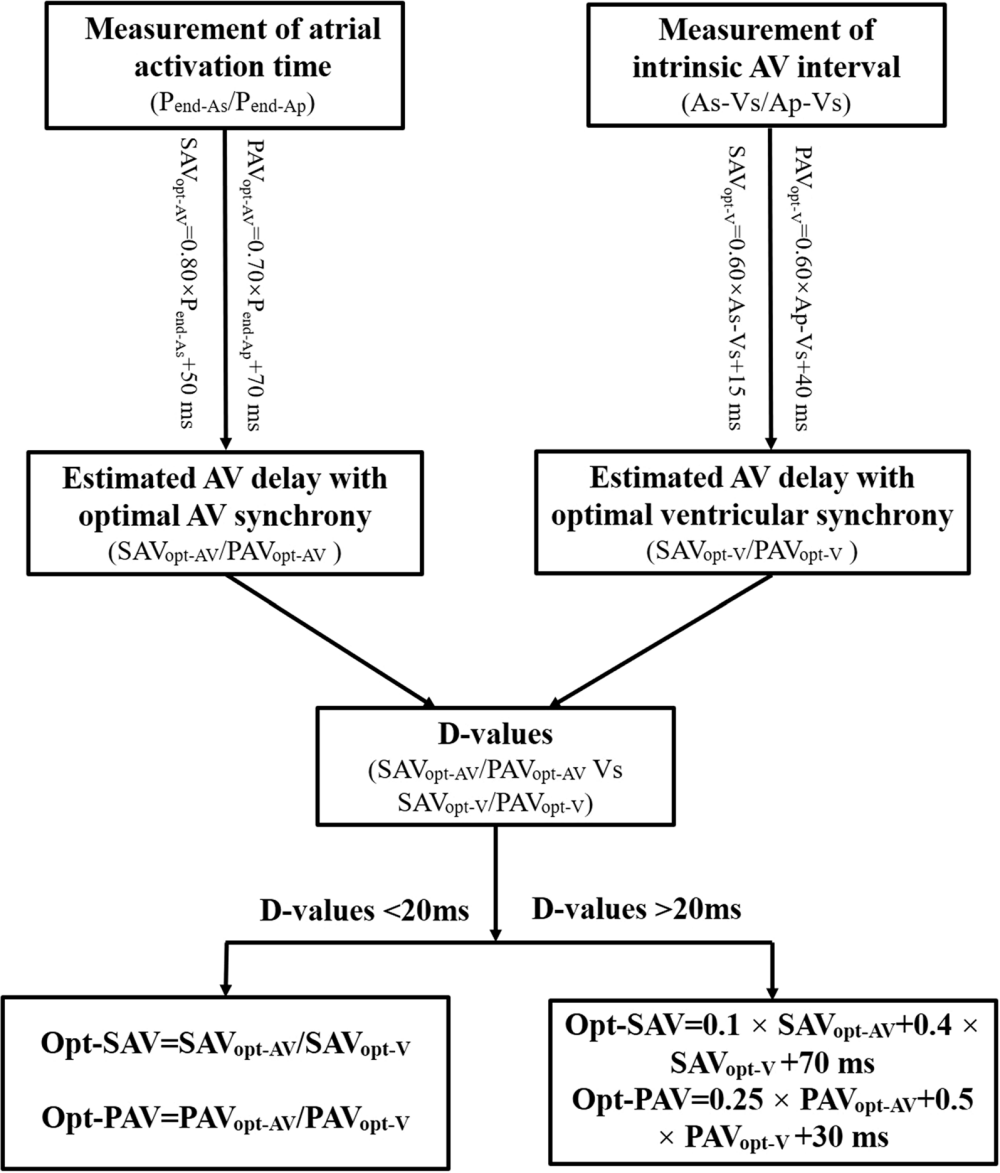
The optimizing algorithm of the optimal AV delay. P_end-As_/ P_end-Ap_: atrial activation time at atrial sensing/ pacing; AV: atrioventricular; As-Vs/Ap-Vs: intrinsic atrioventricular conduction time at atrial sensing/pacing; SAV_opt-AV_/PAV_opt-AV_: sensing/pacing AV delay optimized according to optimal AV synchrony; SAV_opt-V_/PAV_opt-V_: sensing/pacing AV delay optimized according to optimal ventricular synchrony; D-values: different values; opt-SAV/opt-PAV: optimal sensing/pacing AV delay with maximal integrative AV and ventricular resynchronization

AV delay has effects on both AV and ventricular resynchronization simultaneously in CRT, and could be optimized according to the optimal AV and ventricular synchrony respectively. This study showed that SAV_opt-AV_/PAV_opt-AV_ were related to the atrial activation time (P_end-As_/P_end-Ap_), and SAV_opt-V_/PAV_opt-V_ were related to the intrinsic atrioventricular conduction interval (As-Vs/Ap-Vs). However, nearly 50% of patients showed a significant difference between SAV_opt-AV_/PAV_opt-AV_ and SAV_opt-V_/PAV_opt-V_ (D-values > 20 ms). At this time, opt-SAV/opt-PAV optimized according to the maximal AoVTI were linearly correlated with SAV_opt-AV_/PAV_opt-AV_ and SAV_opt-V_/PAV_opt-V_, and had significantly improved haemodynamics. Therefore, the optimal AV delay in CRT could be considered as SAV_opt-AV_/PAV_opt-AV_ or AV_opt-V_/PAV_opt-V_ if the D-values were < 20 ms, or it could be achieved by formulas (opt-SAV = 0.1 × SAV_opt-AV_ + 0.4 × SAV_opt-V_ + 70 ms; opt-PAV = 0.25 × PAV_opt-AV_ + 0.5 × PAV_opt-V_ + 30 ms) if the D-values were > 20 ms. The AV optimized algorithm with the maximal integrative effects of AV and ventricular resynchronization is shown in Fig. 4. In this algorithm, the required parameters were just the atrial activation time (Pend-As/Pend-Ap) and the intrinsic atrioventricular conduction interval (As-Vs/Ap-Vs), which could be measured over several minutes during device interrogation. Therefore, it was easy to perform and special equipment was not required.

Optimal atrioventricular synchrony is a mechanical status in which the onset of LV contraction only coincides with the end of LA contraction and yields the longest diastolic filling time and a fully active filling phase. This is also the principle underlying the Ritter method and its analogues for the optimization of AV delay [9]. Previous studies have shown that this type of AV delay is related to the atrial conduction time [13]. In this study, we defined this AV delay for optimal atrioventricular synchrony as AV_opt-AV_. Using the duration from As/Ap to the relevant end of the P wave (P_end-As_/ P_end-Ap_) as the atrial activation time, the results showed that SAV_opt-AV_/PAV_opt-AV_ were linearly correlated with P_end-As_/P_end-Ap_. Similar results were also found by Jones et al. [14], who suggested that SAV = P_end-As_ + 40 ms and PAV = P_end-Ap_ + 30 ms. We did not find any significant differences when comparing these two equations with our formulas (both *P* > 0.05, data not shown).

The duration of QRS is a marker of ventricular synchrony. Many studies have shown that the extent of a decrease in QRS duration is related to clinical improvement and reverse remodeling of the LV in CRT [7, 15, 16]. Achieving the narrowest QRS duration was first applied in VV optimization [17, 18]. This was further used in AV optimization to maximize the LV global contractile function [12, 13, 19] because the narrowest QRS duration could be obtained by maximal fusion between intrinsic atrioventricular activation and paced activation [6, 7]. Therefore, the AV delay for optimal ventricular synchrony according to the narrowest QRS duration should be correlated with the intrinsic AV conduction interval. In this study, we defined the AV delay for the narrowest QRS as SAV_opt-V_/PAV_opt-V_, and found that they were correlated with intrinsic AV conduction (As-Vs/Ap-Vs).

However, in patients with CHF, the intrinsic AV conduction interval and the atrial activation time are not always proportional. In our patients, we could not find a significant correlation between As-Vs/Ap-Vs and P_end-As_/P_end-Ap_ (data not shown). Therefore, the AV delay, which was optimized according to maximal AV synchrony and was correlated with the atrial activation time, did not always coincide with AV delay optimized according to the maximal ventricular synchrony, which was correlated with intrinsic AV conduction. In this study, no significant correlations were found between SAV_opt-AV_/PAV_opt-AV_ and SAV_opt-V_/PAV_opt-V_, and almost 50% of patients showed > 20-ms differences between SAV_opt-AV_/PAV_opt-AV_ and SAV_opt-V_/PAV_opt-V_. These findings indicated that the AV delay optimized only according to the optimal AV synchrony or ventricular synchrony was not optimal in approximately half of the patients with CRT. In fact, some studies showed that the narrowest QRS complex in CRT was not always associated with the maximal improvement of cardiac contractive function [13]. Sometimes the AV delay optimized by the Ritter method is not as useful as other methods (e.g., Doppler-derived AoVTI) [2, 18, 20]. This further indicates that the optimization of the AV delay only according to either optimal AV or ventricular synchrony is not sufficient in CRT. Therefore, the optimal AV delay with maximal hemodynamics improvement should be the AV interval that produces maximal integration of atrioventricular and ventricular synchrony. In this study, we found that the optimal AV delay with maximal AoVTI was linearly correlated with SAV_opt-AV_/PAV_opt-AV_ and SAV_opt-V_/PAV_opt-V_ when the D-values between them were > 20 ms. The AoVTIs on opt-AV were either significantly greater or not less than those on AV_opt-AV_ and AV_opt-V_. These results indicated that the AV optimization according to the maximal integrative effects of AV and ventricular resynchronization was significantly more effective than the AV interval determined by either optimal AV or ventricular synchrony alone.

There are several limitations to our study. First, we only enrolled 49 patients with CRT. This relatively small number of cases might have affected the accuracy of the regression formula, although the derived equations were statistically significant. Second, the atrial electrodes were all placed in the right atrial appendages in our patients. The sites of the atrial electrodes could affect the measurement of the atrial activation time (As-P_end_/Ap-P_end_) and the intrinsic AV conduction time (As-Vs/Ap-Vs). Therefore, the formulas that were used in our study might not be appropriate for other CRT patients whose atrial electrodes were placed in different sits. Third, the location of the LV lead could affect the QRS fusion pattern with intrinsic atrioventricular activation, and was likely to have significant contributions to the AV_opt-V_. Although LV leads were implanted in the middle or basic segment of lateral/posterolateral veins in majority of our cases (65.3%), it would be better to perform subgroup analysis according to the location of the LV lead and further work is preferable with a sufficient number of cases. Additionally, we only focused on the optimal AV delay in the condition of biventricular simultaneous pacing but did not study the situation of only LV pacing. However, when or how to select the pacing mode of only LV pacing is still controversial. AV and ventricular synchrony must also be considered simultaneously when the AV delay is optimized in only LV pacing mode. Finally, our study was just designed to establish an optimized method with a cross-sectional study, and did not follow the regular visits. Moreover, the detections were performed in patients with stable status of heart failure, which could be seen by the lower using of diuretic in the study cohort. Although the AoVTI was the maximum in the opt-AV, and it is well known that acute hemodynamic improvements measured by echocardiography are related with the outcomes of CRT. The clinical benefit of this algorithm needs further investigation in controlled and prospective studies.

## Conclusions

AV delay could affect atrioventricular and ventricular synchrony in CRT. The AV delay optimized according to the optimal atrioventricular synchrony or optimal ventricular synchrony is correlated with the atrial activation time or the intrinsic AV conduction interval, respectively. However, almost half of the patients showed a significant difference between AV_opt-AV_ and AV_opt-V_. Optimal AV delay is the maximal integration of atrioventricular and ventricular synchrony, and could be considered as SAV_opt-AV_/PAV_opt-AV_ or SAV_opt-V_/PAV_opt-V_ if the D-values were < 20 ms, or could be obtained by formulas that linearly correlated with AV_opt-AV_ and AV_opt-V_ when the D-values were > 20 ms.

## Data Availability

The datasets could be available in the website: https://pan.baidu.com/s/1kQjxnUNu3pgBdGsC9QliiQ, and the enter code: 7qzz.

## Abbreviations

CRT: Cardiac resynchronization therapy
SAV: Sensing atrioventricular delay
PAV: Pacing atrioventricular delay
AV_opt-AV_: AV delay with optimal atrioventricular synchrony
AV_opt-V_: AV delay optimal ventricular synchrony
opt-AV: The optimal AV delay with the maximum integrative effects of AV and ventricular resynchronization
As: Atrial sensing
Ap: Atrial pacing
Vs: Ventricular sensing
As-Vs: The interval from As to Vs
Ap-Vs: The interval from Ap to Vs
P_end-As_: Atrial activation time (duration from As to the end of the P wave)
P_end-Ap_: Atrial activation time (duration from Ap to the end of the P wave)
AoVTI: Aortic velocity–time integral
